# Dangers of Spring

**Published:** 1882-05

**Authors:** 


### DANGERS OF SPRING.

ABOUT one fifth more persons die in New York city in May, than in November. After being pent up in the winter, it might be supposed that the ability to go out and exercise in the luscious air of spring time, would be productive of increased vigor and health of body, but this is simply not the case, as evidenced by the ably prepared and valuable reports.

This difference of mortality between the last month of spring, and the last month of fall, arises from causes which are under the control of the people or beyond; two of each will be mentioned. The natural causes are, 1st. The increased dampness of the asmosphere, proven by the fact that doors which shut easily in winter, do not do so in summer. 2nd. Nature takes away the appetite for meals, for heat giving food, in order to prepare the body for the increased temperature of summer. But two errors in practice at this time, interfere with wise nature's arrangements, and induce many and painful and dangerous diseases. First, the amount of clothing is diminished too soon. Second, the conveniences of fire in our dwellings are removed too early. All persons, especially children, old people, and those in delicate health, should not remove the thickest woolen flannel of mid-winter, nntil sometime in May, and then, it should be merely a change to a little thinner material.

Furnaces should not be removed, nor fire places and grates cleaned for tho summer, until the first of June; for a brisk fire in the grate is sometimes very comfortable in the last week in May; that may be a rare occurrence, but as it does sometimes take place, it is better to be prepared for it, than to sit shivering for half a day, with the risk to ourselves and children, of some violent attack of spring disease. By inattention to these things, four causes are in operation, to chill the body, and induce colds and fevers.

First, The dampness of the atmosphere in May.

Second, The striking falling off in appetite for meats and other "heating" food.

Third, The premature diminution of clothing.

Fourth, The too early removal of the conveniences of fire.

### CLEANLINESS

Of person—the strictest cleanliness—should be among the earliest and most imperative of our teachings to our children; not external cleanliness, but that which is most promotive of health—cleanliness of the skin and the garments which are nearest to it. With what contempt would we look on the best dressed and handsomest person on the street, if we could know that the feet had not been washed for a week, nor the inner garments for a month; and yet it is undeniable that many persons are satisfied that the outer garment should be unexceptionably clean; if that be whole and without a rent, it matters not how soiled and tattered those out of sight are. No such mind can be pure; it implies a deceptiousness of heart which it is impossible to admire. Let mothers especially charge it upon their daughters from earliest life, that it is actually as discreditable to have a hole in the stocking as in the silk dress; that a splotch or stain, or grease spot on an inner garment, is not less unpardonable than if found on a shawl or cloak, or bonnet. Let every mother feel that cleanliness, temperance and thrift, are the antipodes of filth, bestiality and improvidence, and that spotless cleanliness of person, and purity of mind, are absolutely inseparable.

---

### A GOOD WIFE.

In the eighty-fourth year of his age, Dr. Calvin Chapin wrote of his wife: "My domestic enjoyments have been, perhaps, as near perfection as the human condition permits. *She made my home the pleasantest spot to me on earth.* And now that she is gone, my worldly loss is perfect."

How many a poor fellow would be saved from suicide, from the penitentiary and the gallows every year, had he been blessed with such a wife.

"She made home the pleasantest spot to me on earth." What a grand tribute to that woman's love, and piety, and common sense. Rather different was the testimony of an old man some three years ago, just before he was hung in the Tombs' yard of this city. "I didn't intend to kill my wife, but she was a very aggravating woman." Let each wife inquire, "Which am I?"

